# Narirutin treatment accelerates the process of diabetic wound repair by regulating phenotype switching of macrophages through affecting metabolic reprogramming

**DOI:** 10.3389/fphar.2025.1614967

**Published:** 2025-06-30

**Authors:** Liang Liu, Han Wang, Juan Zhou, Jing Lu, Yan Zhang, Haiyan Liu, Li Lu

**Affiliations:** ^1^ Department of Cardiology, Hubei Provincial Hospital of Traditional Chinese Medicine, Wuhan, China; ^2^ Department of Anesthesiology, Peking University People’s Hospital, Qingdao, China; ^3^ Department of Anesthesiology, Women and Children’s Hospital, Qingdao University, Qingdao, China; ^4^ Department of Rehabilitation, Tongji Hospital, Tongji Medical College, Huazhong University of Science and Technology, Wuhan, China

**Keywords:** diabetic foot ulcer, narirutin, macrophage, metabolic reprogramming, AMPK, inflammation

## Abstract

**Introduction:**

Diabetic foot ulcer (DFU) is one of the most common complications of diabetes, with substantial morbidity and mortality. Narirutin (Nar), a bioactive phytochemical derived from citrus peel, has been suggested to possess anti-inflammatory abilities. However, the involvement of Nar in DFU development remains poorly understood.

**Methods:**

The polarization traits of bone marrow derived macrophages (BMDMs) with indicated treatments were determined by flow cytometry, immunofluorescence staining, western blot and qRT-PCR. Levels of lactate and α-ketoglutarate were measured for investigating the metabolic profiles. The cutaneous wounds of diabetic mice were established for evaluating the promotive roles of Nar in wound healing *in vivo.*

**Results:**

We found that high glucose treatment significant elevated the contents of TNF-α and IL-1β and lactate and reduced the levels of TGF-β1 and IL-4 and α-ketoglutarate in BMDMs. Then, Nar intervention effectively induced BMDMs repolarization from M1 to M2 state and the molecular mechanism was ascribed to drug-elicited activation of AMPK, which in turn increased the expression of downstream Mfn2, thereby enhancing the activity of oxidative phosphorylation and GATA3 cascade activation and disrupting the progress of glycolysis and NF-κB axis activation. Subsequently, we discovered that Nar injection effectively enhanced the healing rate of skin wounds in diabetic mice. Histological analysis showed that Nar dose-dependently induced dermis growth and collagen deposition in the wound area. Via activating AMPK/Mfn2 axis, Nar inhibited the activity of glycolysis and enhanced the extent of oxidative phosphorylation, accompanied by inflammation repression and angiogenesis promotion in the damaged tissue

**Discussion:**

Our study discovered that macrophages repolarization to M2 phenotype was required for Nar-induced promotive effects on diabetic wound repair by regulating reprogramming of glucose metabolism via mediating AMPK/Mfn2 pathway, providing a promising strategy for DFU management.

## 1 Introduction

Epidemiological data have established that the global incidence of diabetes is increasing year by year, with nearly 30% of patients developing diabetic foot ulcer (DFU), which is associated with poor prognosis and a high death rate (Armstrong et al., 2023). Approximately one-third of individuals with a DFU will experience a severe infection and even a lower extremity amputation, which puts a huge burden on the social economy and health (Evstratova et al., 2024; Spampina et al., 2020). Due to the limited effects produced by current treatment modalities like surgical debridement, antibacterial dressing, and biomaterial applications, seeking alterative and complementary agents with high efficiency and safety to antagonize DFU is urgent (Li et al., 2023; Petric et al., 2025; Szunerits et al., 2025).

Typically, the healing process of acute skin wounds can be divided into four sequential steps: hemostasis, inflammation, proliferation, and remodeling. It has been shown that macrophages play pivotal roles during the course of wound repair (Mao K. et al., 2025). In the inflammation stage, under the stimulation of pro-inflammatory factors, macrophages are recruited to the damaged area where they phagocytize invaded pathogens and cellular debris and secrete multiple cytokines to augment inflammation responses for accelerating the elimination of exogenous microbes and injured tissues. Then, at the proliferation phase, macrophages are transformed from M1 to the anti-inflammatory M2 phenotype for confining the inflammation extent and releasing diverse growth factors, thereby enhancing the process of tissue repair (Xiong et al., 2023; Li et al., 2021). However, in the diabetic microenvironment, several pathological factors, like hyperglycemia, hypoxia, and pH abnormality, hamper the M2 phenotype repolarization of macrophages, which cause local inflammation exacerbation and pro-proliferative cytokine deficiency, ultimately leading to angiogenesis dysfunction and delayed healing of diabetic wounds (Wu et al., 2022).

It has been shown that metabolites *in vivo* are closely associated with the processes of immune regulation (Zhu et al., 2021). Lactate, as a main product from the glycolytic pathway, is reported to participate in the pro-inflammatory reactions of M1 macrophages (Feng et al., 2022). α-Ketoglutarate (α-KG) is an intermediate metabolite of oxidative phosphorylation (OXPHOS) and acts as a pivotal anti-inflammatory effector in M2 macrophages (Zhou et al., 2022). There is evidence that activity changes in the metabolic pathways like inhibition of glycolysis and enhancement of oxidative phosphorylation, which are termed as metabolic reprogramming, are indicated to be implicated in the inflammation resolution of macrophages (Faas et al., 2021). Previous studies have shown that AMP-activated protein kinase (AMPK) serves as an important intracellular energy sensor responsible for the anabolism and catabolism of glucose and lipid. Mitofusin 2 (Mfn2) locates in the mitochondrial outer membrane and takes part in the regulation of mitochondria fusion and fission to maintain physiological functions of the organelle (Zhang et al., 2021). Isolated from citrus fruits, narirutin (Nar) is found to be a bioactive phytochemical possessing potent anti-inflammatory and anti-oxidative capacities (Luo et al., 2024; Gao et al., 2025). It was uncovered that Nar restrains NLRP3 inflammasome assembly and IL-1β generation of macrophages via abrogating NF-κB, MAPK, and PI3K/Akt cascades (Ri et al., 2023). To date, whether Nar suppresses the inflammation development in macrophages through regulating metabolic reprogramming remains unclear. In this study, we discovered that Nar effectively strengthened oxidative phosphorylation and encumbered glycolysis by activating the AMPK/Mfn2 signaling axis, thereby increasing α-KG levels and reducing lactate, which were accompanied by the alleviation of inflammation in macrophages and enhanced angiogenesis.

## 2 Materials and methods

### 2.1 Reagents

Nar (purity >99.8%) was obtained from MedChemExpress (Monmouth Junction, NJ, United States) and was dissolved in dimethyl sulfoxide (DMSO, St. Louis, MO, United States). Cell Counting Kit-8 was purchased from Boster Biological Technology Co., Ltd. (Wuhan, China). The calcein/PI cell viability/cytotoxicity assay kit and the 5-ethynyl-2′-deoxyuridine (EdU) cell proliferation kit were acquired from Beyotime (Shanghai, China). Flow cytometry anti-CD11b (FITC), anti-F4/80 (APC), anti-CD86 (PE), and anti-CD206 (PerCP) were purchased from BioLegend, Inc. (San Diego, CA, United States). Anhydrous D-glucose was obtained from Solarbio Science & Technology Co., Ltd. (Beijing, China). The AMPK inhibitor Compound C was acquired from Selleck (Houston, Texas, United States). The reagent for blood biochemistry was purchased from Nanjing Jiancheng Bioengineering Institute (Nanjing, China). The Lactic Acid Content Assay Kit and the α-KG Content Assay Kit were purchased from Solarbio (Beijing, China). The Lipofectamine 3000 kit was obtained from Life Technologies (Carlsbad, CA, United States). Matrigel Matrix was purchased from Corning, Inc. (NY, United States). Primary antibodies for arginase 1 (Arg-1), inducible nitric oxide synthase (iNOS), TNF-α, TGF-β1, AMPK, p-AMPK, Mfn2, carnitine palmitoyl-transferase 1A (CPT-1A), isocitrate dehydrogenase 3 (IDH3), lactate dehydrogenase (LDH), p65, p-p65, and GATA binding protein 3 (GATA3) were obtained from Cell Signaling Technology (Danvers, MA, United States). Primary antibodies for CD86, CD206, proliferating cell nuclear antigen (PCNA), p21, matrix metalloproteinase-9 (MMP-9), tissue inhibitor of metal protease 1 (TIMP-1), vascular endothelial growth factor receptor 2 (VEGFR2), PI3K, p-PI3K, Akt, p-Akt, hypoxia-inducible factor-1α (HIF-1α), cytochrome c oxidase subunit 4 (COX IV), and GAPDH were purchased from ABclonal (Wuhan, China). The reverse transcription kit and SYBR-Green PCR Master Mix were purchased from Vazyme Biotech Co., Ltd. (Nanjing, China).

### 2.2 Cell culture

Bone marrow-derived macrophages (BMDMs) were acquired as previously reported (Li et al., 2019). In brief, the femur and tibia were obtained from healthy male C57BL/6 mice aged 6 weeks. Bone marrow cells were collected by rinsing the marrow cavity, and they were resuspended in RPMI-1640 supplemented with 10% fetal bovine serum and 40 ng/mL murine recombinant M-CSF. After being cultured for 7 days in a 37°C incubator with 5% CO_2_, the cells differentiated into BMDMs, which were then used for the following experiments.

Human umbilical vein endothelial cells (HUVECs) were obtained from the American Type Culture Collection (Rockville, MD, United States) and maintained in RPMI-1640 containing 10% fetal bovine serum. The culture medium was renewed every 2 days, and cells at the logarithmic growth stage were collected for *in vitro* experiments.

### 2.3 CCK-8 assay

The Cell Counting Kit-8 was applied to detect the viability of BMDMs in accordance with the manufacturer’s protocols. The BMDMs were seeded into a 96-well plate at a density of approximately 6 × 10^3^ cells per well. Then, they were co-incubated with different concentrations of Nar (0, 50, 100, 200, and 400 μM) for a duration of 24 h. A volume of 10 μL CCK-8 dye solution was added to each well, and the plate was maintained at 37°C for 2–4 h. The absorbance value at a wavelength of 450 nm was measured using a microplate reader.

### 2.4 Live/dead staining

To further determine the cytotoxicity of Nar on BMDMs, cells were co-cultured with Nar in a 96-well plate for 24 h. Then, the samples were washed with PBS and stained with calcein AM and PI for 30 min at 37°C in the dark. The images were captured using a fluorescence microscope.

### 2.5 Flow cytometry

BMDMs were distributed into individual wells of a 6-well plate and exposed to varying concentrations of high glucose (HG) (0, 15, 30, and 45 mM) with/without incubation with Nar for 24 h. Subsequently, cells were collected, washed with ice-cold PBS, and treated with an unlabeled anti-CD16/32 antibody to block Fcγ3 peptide. Cells were then stained with CD11b, F4/80, CD86, and CD206 diluted in FACS buffer for 30 min at 4°C. Samples were acquired using the LSRFortessa X-20 cytometer, and data analyses were performed using FlowJo software.

### 2.6 RNA interference assay

The small interfering RNA (siRNA) sequences targeting Mfn2 were synthesized by GenePharma (Shanghai, China). BMDMs were cultured into a 6-well plate at a concentration of 10^6^ per well. Then, Lipofectamine 3000 and opti-minimum essential medium were used for transfection. The Mfn2 siRNA sequences were shown as follows: Mfn2-siRNA Forward (5′−3′) GCG​GGU​UUA​UUG​UCU​AGA​AAU​TT, Reverse (5′−3′) AUU​UCU​AGA​CAA​UAA​ACC​CGC​TT.

### 2.7 The 5-ethynyl-2′-deoxyuridine incorporation assay

HUVECs were seeded into a 24-well plate and co-incubated with BMDMs pretreated with varying dosages of HG. Then, EdU working solution was added to each well for 2 h. The HUVECs were washed with PBS and fixed with 4% paraformaldehyde for 30 min. Subsequently, the nuclei were stained with Hoechst33342 for 30 min. After being washed thrice, the cells were examined under a fluorescence microscope.

### 2.8 Cell scratch test

HUVECs were cultured into the lower chamber of a 6-well plate and scratched with a sterile 1-mL pipette tip. Then, the cells were exposed to the stimulation of BMDMs pretreated with HG in the upper chamber. After 24 h of incubation, HUVECs were photographed, and the remaining wound area was measured using ImageJ software.

### 2.9 Transwell migration assay

The 24-well culture plates inserted with 8-μm-pore-sized filters were used to evaluate the cell migratory ability. HUVECs (1 × 10^4^) were suspended in low-serum (5% fetal bovine serum) medium and plated into the upper chamber. BMDMs suspended in complete medium (10% fetal bovine serum) were seeded into the lower chamber. After being cultured for 24 h, HUVECs migrated on the bottom side of the filter were fixed and stained with 0.5% crystal violet, and then they were captured using an optical microscope.

### 2.10 Tube formation assay

For the tube formation detection, 250 μL of cold Matrigel was added to each well of a 24-well plate and maintained at 37°C for 1 h. Subsequently, HUVECs (2 × 10^4^ per well) were plated on the Matrigel-coated plate and co-incubated with BMDMs in the upper chamber for 24 h. The tube length and total branch points were observed using an inverted microscope and assessed using ImageJ software.

### 2.11 Animal experiment

Male C57BL/6 mice aged 6–8 weeks were obtained from Vital River Laboratory Animal Technology Co. Ltd. (Beijing, China). Animals were maintained under specific pathogen-free conditions with a 12-h light–dark cycle, and food and water were provided ad libitum. The mice were fed with a high-fat diet for 4 weeks, accompanied by intraperitoneal administration of streptozotocin (60 mg/kg/day) for 5 days to establish the diabetes model. After the concentration of the random blood glucose exceeded 16.8 mmol/L in two measurements, the mice were selected for the next experiment. Mice were then anesthetized by intraperitoneal injection of pentobarbital sodium (50 mg/kg), and full-thickness excisional skin wounds were made on the dorsum. The animals were randomly allocated and marked with noninvasive skin dying (Klabukov et al., 2023; Sensini et al., 2020) and then received a total of 100 μL PBS, low-dosage Nar (60 mg/kg in 100 μL PBS) or high-dosage Nar (120 mg/kg in 100 μL PBS) injection subcutaneously in four sites adjacent to the wound. After injection, the wounds were covered with transparent dressings (Tegaderm™; 3M™; United States) and photographed at days 0, 3, 7, 10, and 14 post-wounding. The area of wound closure was assessed using ImageJ software. On day 14, mice were sacrificed, and the skin tissues and internal organs were harvested for further analysis. All animal studies were approved by the Institutional Animal Care and Use Committee of the Tongji Medical College, the Huazhong University of Science and Technology (IACUC number: 3936), and studies were performed in accordance with the National Institutes of Health Guide for Care and Use of Laboratory Animals.

### 2.12 Blood perfusion determination in the wound site

On day 10 post-operation, the laser speckle contrast imaging system was used to measure the local blood flow in the wound region. A near-infrared laser at 785 nm was applied to detect blood perfusion, which was termed as the perfusion unit. The wounds were imaged at a fixed distance, and the mean perfusion unit (MPU) ratio was calculated by comparing the MPU of the wound area with that of the area surrounding the wound.

### 2.13 Histopathological analysis

On day 14, wound tissue samples were collected, fixed in 4% paraformaldehyde, and then dehydrated and embedded in paraffin. The paraffin samples were cut into 4-μm-thick sections, which were subjected to hematoxylin−eosin (HE)/MASSON staining as previously described. For immunohistochemistry, the skin wound sections were rehydrated and incubated with antibody against CD31. Subsequently, the sections were incubated with secondary antibody and visualized using the DAB substrate under an optical microscope.

### 2.14 Blood biochemical test

The blood samples of mice were collected on day 14, which were then centrifuged, and the upper serum was obtained. The levels of AST, ALT, and Cr were detected using kits from Nanjing Jiancheng Bioengineering Institute, according to the manufacturer’s protocols.

### 2.15 Immunofluorescence staining

The fixed BMDMs were permeabilized with 0.3% Triton X-100, blocked in 1% BSA, and then incubated overnight with anti-Arg-1 and anti-iNOS at 4°C. The samples were washed with PBS thrice and stained with fluorescence-conjugated secondary antibodies. The nuclei were incubated with DAPI, and the images were captured using a fluorescence microscope.

### 2.16 Quantitative real-time PCR

Total RNA was extracted from BMDMs, HUVECs, and skin wound tissues using TRIzol reagent. The HiScript III RT Super-Mix was used to reverse-transcribe the RNA into cDNA, following the manufacturer’s instructions. Real-time PCR was executed using a Light-Cycler 480 II with 2x SYBR Green qPCR Mix. The 2^−ΔΔCT^ method was used to quantify relative mRNA expression, and GAPDH was used to normalize mRNA levels. Primer sequences are listed in Table 1.

**TABLE 1:** Primer sequences for qRT-PCR

Gene	Forward	Reverse
Arg-1	AGC​CAG​GGA​CTG​ACT​ACC​TT	TTG​GGA​GGA​GAA​GGC​GTT​TG
iNOS	CTG​CCA​GGG​TCA​CAA​CTT​TAC​A	AAC​AGC​TCA​GTC​CCT​TCA​CC
TNF-α	CCC​ACG​TCG​TAG​CAA​ACC​AC	GCA​GCC​TTG​TCC​CTT​GAA​GA
IL-1β	TGC​CAC​CTT​TTG​ACA​GTG​ATG	AAG​GTC​CAC​GGG​AAA​GAC​AC
TGF-β1	ACT​GGA​GTT​GTA​CGG​CAG​TG	GGG​GCT​GAT​CCC​GTT​GAT​TT
IL-4	CCC​CCA​GCT​AGT​TGT​CAT​CC	AGG​ACG​TTT​GGC​ACA​TCC​AT
p21	GGA​GGA​GCA​TGA​ATG​GAG​ACA	AAA​GTT​CCA​CCG​TTC​TCG​GG
PCNA	AGA​TGC​CGT​CGG​GTG​AAT​TT	TGT​TCC​CAT​TGC​CAA​GCT​CT
HIF-1α	TTC​CCC​CGT​CCA​CCC​ATT​TC	GAC​TCT​TTG​CTT​CGC​CGA​GA
MMP-1	AGA​AGT​GTG​ACC​CAG​CCC​TA	GGT​CAC​GGG​ATG​GAT​GTT​CA
MMP-9	ACC​TCC​AAC​CTC​ACG​GAC​A	AGG​TTT​GGA​ATC​GAC​CCA​CG
TIMP-1	AGA​AAT​CAA​CGA​GAC​CAC​CTT​A	TGA​TCC​GTC​CAC​AAA​CAG​TGA
LDH	CTG​TGA​GCC​TGC​TGC​ATT​CG	ACT​TGG​GTG​GTT​GGT​TCC​AT
CPT-1A	CCT​ACA​TGG​GCC​AGG​CTT​TG	GTA​GAC​GTC​CAG​CCA​CAG​TC
IDH3	TCG​AGT​ACG​CTC​GGA​ACA​AC	ACA​GTT​CTC​CGC​AAC​TTC​CC
SDH	TAC​GAG​CAA​CCA​AGT​GGG​AG	AAG​AGG​TTG​AAA​TCG​GGG​CA
AMPK	ACA​CCA​ATT​GAC​AGG​CCA​TAA	AGG​TGC​TGC​ATA​ATT​TGG​CG
Mfn2	AGG​TTG​AGG​TGA​CAG​CGT​TC	GGA​CTC​GAG​GTC​TCC​TCT​GT

### 2.17 Measurement of metabolite lactate and α-KG

After treatment, BMDMs and wound tissues were collected and used for detecting the quantity of endogenous lactate and α-KG using the Lactic Acid Content Assay Kit and α-Ketoglutaric Acid Content Assay Kit, according to the manufacturer’s protocols.

### 2.18 Western blot

The total proteins and mitochondrial proteins from cells and tissues were extracted using kits from Beyotime, according to the protocols. After quantification, protein samples were separated using 10% SDS-PAGE and then transferred onto polyvinylidene difluoride membranes. Then, membranes were blocked with 5% BSA at room temperature for 1 h, followed by incubation with primary antibodies at 4°C overnight. Subsequently, membranes were washed thrice with Tris-buffered saline−Tween 20 and incubated with corresponding secondary antibodies for 1 h at room temperature. The protein bands were visualized using the electrochemiluminescence kit, and the gray values of the bands were quantified and analyzed using ImageJ software.

### 2.19 Statistical analysis

The data in this study were presented as the mean ± standard deviation (SD) and were analyzed using GraphPad Prism software (version 8.0.1; San Diego, CA, United States). Statistical analyses of multiple groups were accomplished using one-way ANOVA, followed by Tukey’s *post hoc* test. P-values < 0.05 were considered statistically significant.

## 3 Results

### 3.1 HG stimulated inflammatory responses and altered the activities of glycolysis and OXPHOS

**FIGURE 1:**
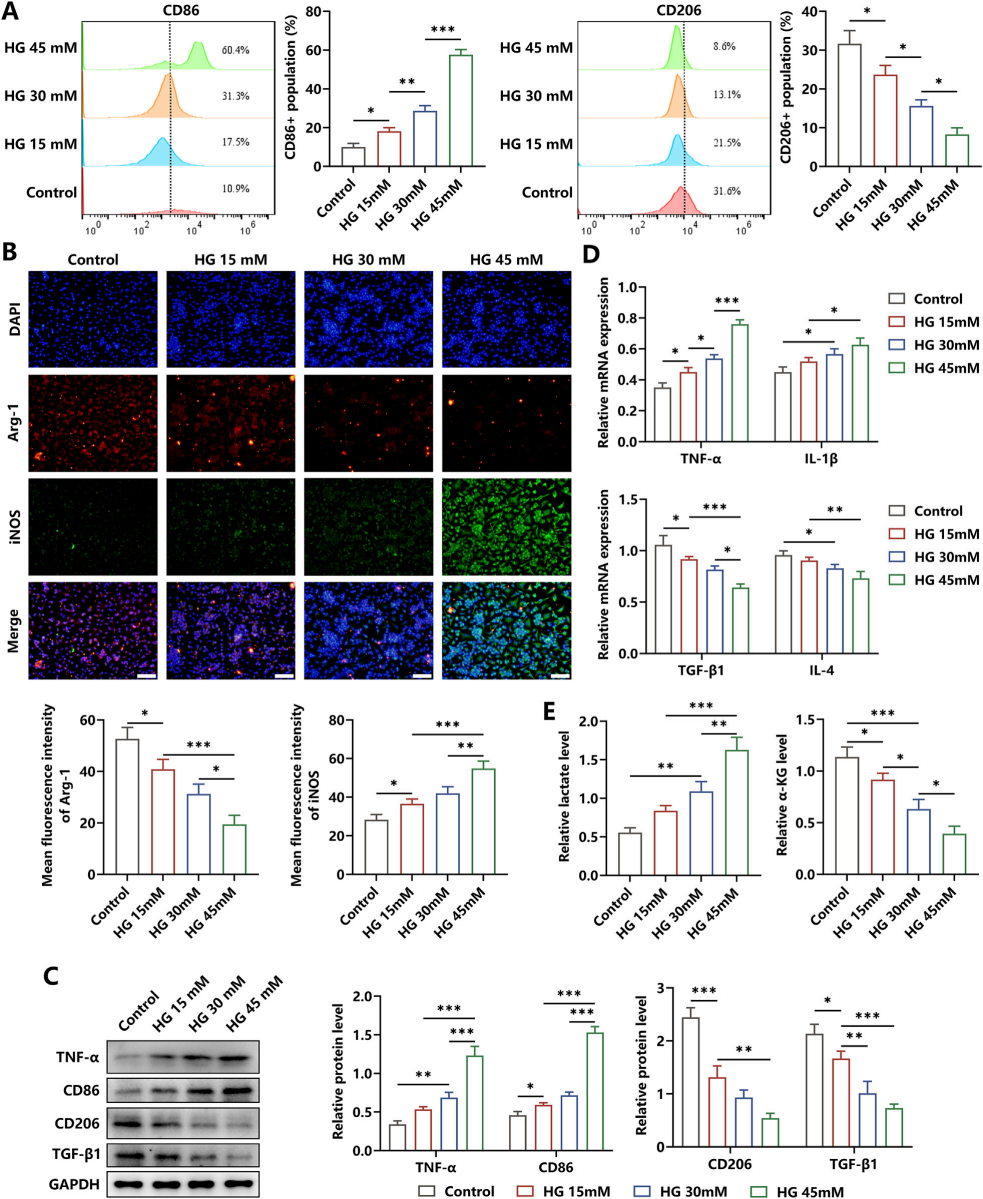
Effects of HG on the inflammation responses in macrophages. **(A)** Flow cytometry analysis showed the phenotype profiles of macrophages induced by different dosages of HG. **(B)** The levels of Arg-1 and iNOS in HG-stimulated BMDMs were determined through immunofluorescence staining (scale bar: 200 μm). **(C)** Western blot analysis showed that there was an increase in the levels of TNF-α and CD86 and a reduction in the contents of CD206 and TGF-β in the macrophages triggered by HG in a dose-dependent manner. **(D)** Relative mRNA expressions of pro-inflammatory factors and anti-inflammatory biomarkers. **(E)** HG dose-dependently increased the level of lactate and decreased the level of α-KG in macrophages. The experiments were performed independently thrice. *p < 0.05, **p < 0.01, and ***p < 0.001.

To observe the effects of the high-glucose microenvironment on regulating the inflammatory responses in macrophages, we treated the BMDMs with HG for 24 h and found that the percentage of the M1 phenotype was increased and the proportion of the M2 phenotype was reduced, as indicated by elevated levels of CD86 and iNOS and decreased levels of CD206 and Arg-1 (Figures 1A–C). Then, we discovered that the pro-inflammatory cytokines TNF-α and IL-1β showed increased concentrations, whereas the anti-inflammatory cytokines TGF-β and IL-4 showed reduced contents in BMDMs under HG stimulation (Figures 1C,D). In addition, the level of metabolite lactate in the glycolysis pathway was decreased, and the content of metabolite α-KG in the OXPHOS was increased in HG-irritated BMDMs in a dose-dependent manner (Figure 1E). These results showed that HG administration significantly induced macrophages to polarize to the M2 status, accompanied by the inflammation response progression.

### 3.2 Narirutin induced phenotype repolarization of macrophages stimulated by HG

**FIGURE 2:**
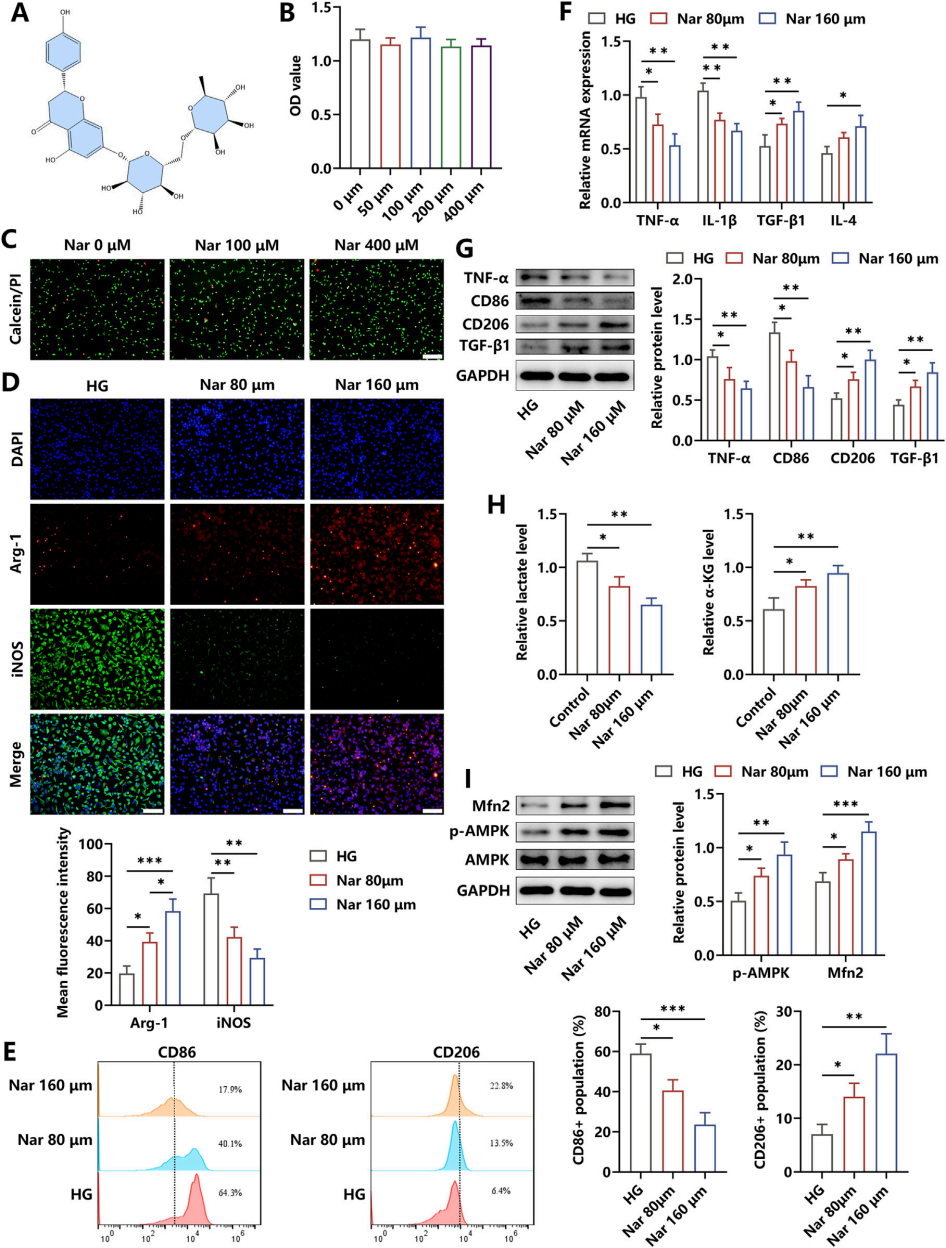
Nar treatment effectively attenuated inflammation in HG-stimulated macrophages. **(A)** Chemical structure of Nar. **(B,C)** Effects of Nar on viability of BMDMs were detected using the CCK-8 test and calcein/PI staining (scale bar: 200 μm). **(D)** BMDMs were incubated with HG (45 mM) in the presence of Nar with different doses (0, 80, and 160 μM) for 24 h. The levels of Arg-1 and iNOS in each group were detected through immunofluorescence staining (scale bar: 200 μm). **(E)** Flow cytometry analysis of macrophages with indicated treatments. **(F)** The results of qRT-PCR showed that Nar reduced the mRNA expression of pro-inflammatory factors and elevated the mRNA expression of anti-inflammatory molecules. **(G)** Nar dose-dependently decreased the protein levels of TNF-α and CD86 and elevated the protein contents of CD206 and TGF-β in macrophages. **(H)** The production of lactate and α-KG in BMDMs was reduced and increased separately after Nar treatment in a dose-dependent fashion. **(I)** AMPK phosphorylation and Mfn2 expression were evaluated using Western blot; n = 3; *p < 0.05, **p < 0.01, and ***p < 0.001.

The chemical structure of Nar, which included flavanone naringenin bound to the disaccharide rutinose, is shown in Figure 2A. We evaluated the cytotoxicity of Nar and found that the viability of BMDMs was unchanged after the incubation with varying dosages of Nar (0–400 μM) for 24 h, as indicated by the results of the CCK-8 test and calcein AM/PI staining (Figures 2B,C). To investigate the regulatory roles of Nar in the inflammatory status of macrophage, Nar was administered to BMDMs with simultaneous HG incubation for 24 h. Our findings revealed that different concentrations of Nar promoted M1 macrophage repolarization to the M2 phenotype, as evidenced by the reduced expressions of iNOS, CD86, TNF-α, and IL-1β and increased levels of Arg-1, CD206, TGF-β, and IL-4 (Figures 2D–F). Results of Western blot indicated that HG-triggered elevation in TNF-α and CD86 levels and reduction in TGF-β and CD206 contents were reversed by Nar intervention in a dose-dependent way in BMDMs (Figure 2G). Then, we observed that Nar significantly alleviated HG-induced increase in lactate content and decrease in α-KG content (Figure 2H). Moreover, AMPK participating in glucose metabolism was observed to possess increased activity in the presence of Nar treatment. The decreased expression of Mfn2, which was involved in mitochondrial homeostasis in HG-primed macrophages, was enhanced by Nar administration (Figure 2I). Our results suggested that Nar treatment effectively weakened inflammation responses and affected glucose metabolism pathways in macrophages.

### 3.3 Nar induced metabolic reprogramming to trigger inflammation amelioration

**FIGURE 3:**
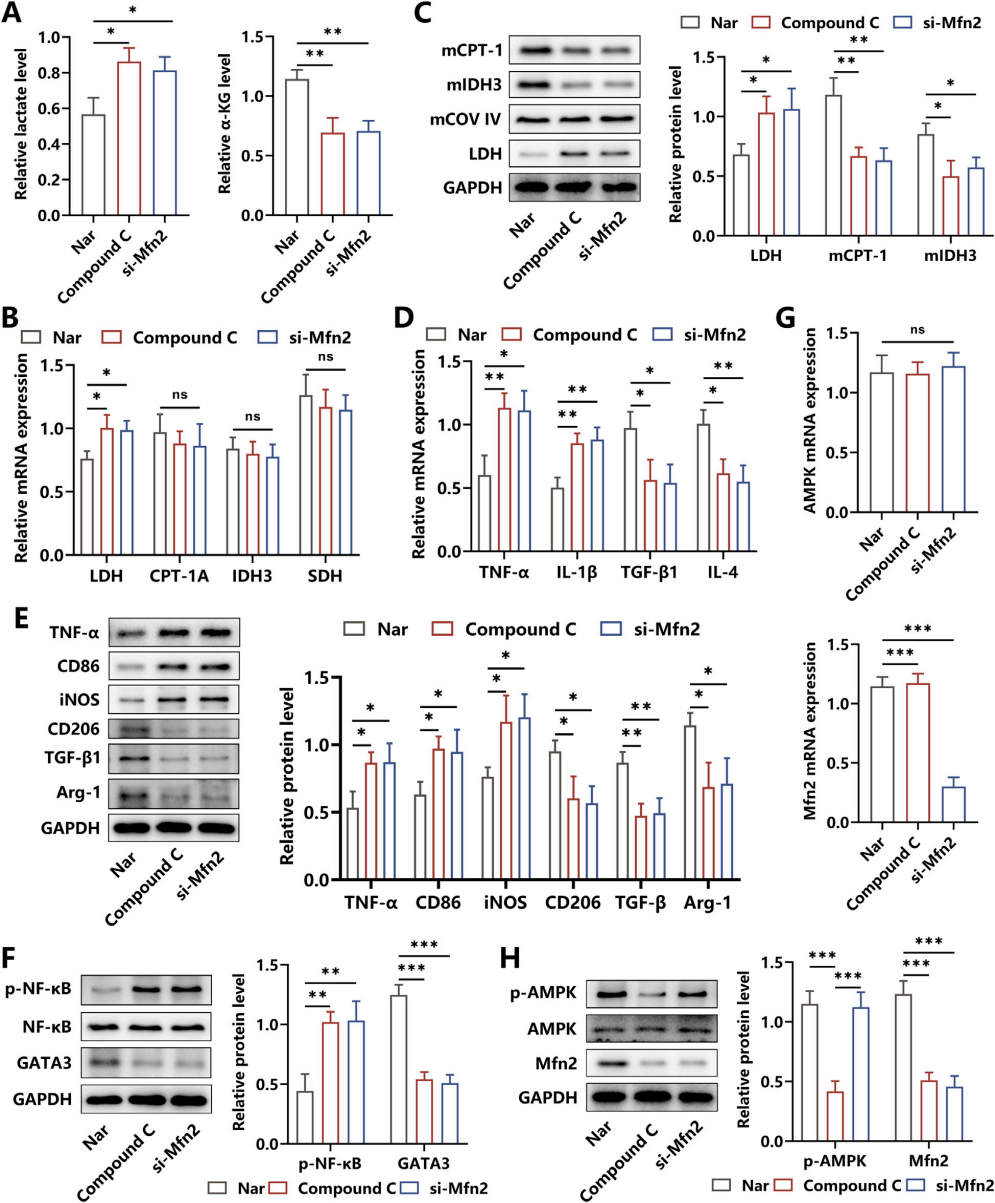
Effects of AMPK inhibition and Mfn2 silencing on the glucose metabolism and inflammation response of Nar-treated macrophages. **(A)** Both HG (45 mM) and Nar (160 μM) were incubated with BMDMs in the presence of PBS, Compound C, or si-Mfn2, respectively, for 24 h. Relative levels of lactate and α-ketoglutaric acid in each group. **(B)** The mRNA expressions of LDH, CPT-1A, IDH3, and SDH were determined using qRT-PCR. **(C)** The protein levels of LDH, mitochondrial CPT-1A, and mitochondrial IDH3 were detected using Western blot. **(D)** Results from qRT-PCR showed that Compound C or si-Mfn2 intervention reversed Nar-induced suppression of TNF-α and IL-1β levels and inhibited Nar-triggered elevation of TGF-β and IL-4 levels in macrophages. **(E)** Western blot analysis showed that the protein levels of M1-related biomarkers and M2-related molecules were increased and reduced separately in BMDMs after Compound C or si-Mfn2 administration. **(F)** The activities of NF-κB and GATA3 expressions were measured using Western blot. **(G,H)** The expressions of AMPK and Mfn2 at the transcriptional and translational levels were detected using qRT-PCR and Western blot, respectively; n = 3; *p < 0.05, **p < 0.01, and ***p < 0.001.

It was illustrated that Mfn2 displayed crucial roles in maintaining the biological functions of mitochondrion, and AMPK was reported to be an upstream regulator of Mfn2, participating in multiple pathophysiological processes (Zhang et al., 2021). Herein, to assess whether Nar altered the glucose metabolism pathway by regulating the AMPK/Mfn2 signal cascade, the BMDMs were co-incubated with Nar and HG simultaneously in the absence/presence of Compound C or si-Mfn2 for 24 h. We discovered that both Compound C and si-Mfn2 administration abolished Nar-induced reduction in lactate levels and elevation of α-KG content in macrophages (Figure 3A). Then, LDH responsible for the glycolysis process was found to be upregulated after the intervention of Compound C or si-Mfn2. Of note, the expressions of mitochondrial CPT-1A, IDH3, and succinate dehydrogenase (SDH) acting as rate-limiting enzymes of the OXPHOS process were observed to be unchanged in Nar-induced macrophages supplemented with Compound C or si-Mfn2 treatment at the transcriptional level but were significantly suppressed at the translational level (Figures 3B,C).

Considering that glycolysis and OXPHOS dominate the glucose metabolic pathway in M1 and M2 phenotypes of macrophages, respectively, strategies targeting metabolic reprogramming had been proven to be efficient in regulating inflammation responses in macrophage (Faas et al., 2021). Then, to probe the effects of the AMPK/Mfn2 axis-related alteration in metabolic pathways on Nar-evoked inflammation relief in macrophages, the key cytokines and inflammatory signaling cascades were detected. As shown in Figures 3D,E, after the administration of Compound C or si-Mfn2, the contents of TNF-α and IL-1β were increased and the levels of TGF-β and IL-4 were decreased, accompanied by the elevated expressions of M1 biomarkers CD86 and iNOS and reduced expressions of M2 biomarkers CD206 and Arg-1. Additionally, our results indicated that the increased activity of the pro-inflammatory signal factor NF-κB and decreased expression of the anti-inflammatory signal molecule GATA3 were observed in Compound C- or si-Mfn2-treated BMDMs (Figure 3F). We further determined the interaction between AMPK and Mfn2 and discovered that Compound C intervention suppressed the expression of Mfn2 at both the transcriptional and translational levels, whereas si-Mfn2 failed to alter the expression and the activity of AMPK, indicating the upstream regulatory roles of AMPK in Mfn2 expression (Figures 3G,H). Considering the abovementioned results, we had reasons to believe that the mechanisms underlying Nar-elicited anti-inflammatory effects were to some extent attributed to the metabolic reprogramming induced by the AMPK/Mfn2 signal cascade.

### 3.4 Nar-induced anti-inflammatory effects improved the pro-angiogenic abilities of HUVECs

**FIGURE 4:**
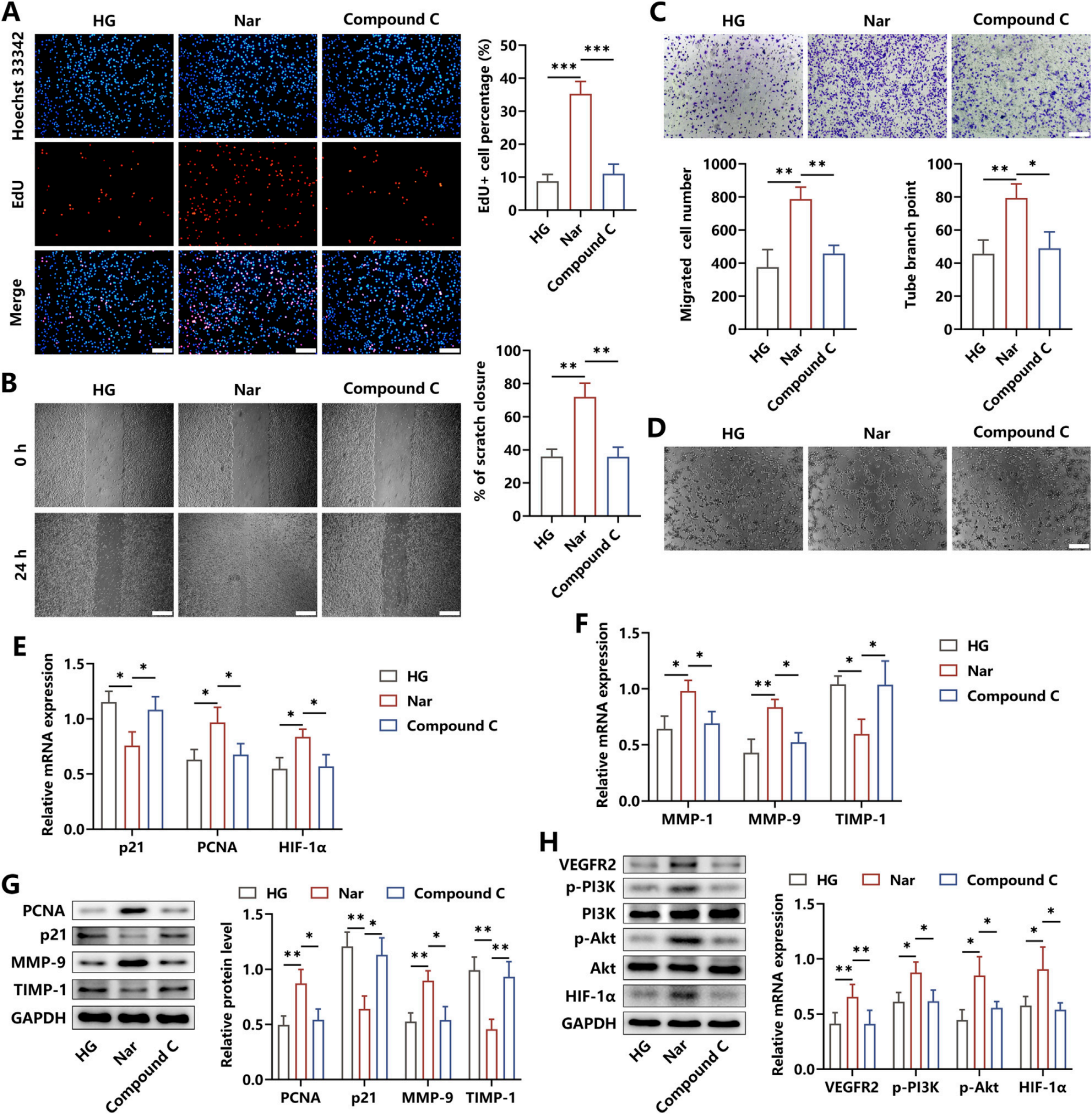
AMPK signaling affected the roles of macrophage-produced micro-niche in regulating angiogenic abilities of HUVECs. **(A)** BMDMs were incubated with HG, HG + Nar, or HG + Nar + Compound C for 24 h. Afterward, the cells were seeded in the upper chamber, and HUVECs were seeded in the lower chamber for 24 h. Edu staining showed that Compound C reversed Nar-induced proliferation enhancement of HUVECs (scale bar: 200 μm). **(B, C)** Results of cell scratch and transwell migration assay indicated that the increased migration of HUVECs induced by Nar was repressed by Compound C (scale bar: 500 μm **(B)** and 200 μm **(C)**). **(D)** Nar treatment enhanced the tube formation ability of HUVECs, which were inhibited by Compound C (scale bar: 200 μm). **(E, F)** The mRNA expression of angiogenesis-related genes was detected using qRT-PCR. **(G)** The protein levels of PCNA, p21, MMP-9, and TIMP-1 were measured using Western blot. **(H)** Molecular expression of the VEGFR2/PI3K/Akt/HIF-1α axis was determined using Western blot; n = 3; *p < 0.05, **p < 0.01, and ***p < 0.001.

It has been demonstrated that inflammation acts as a pivotal pathogenic factor antagonizing the development of angiogenesis. To verify whether Nar alleviated the inhibitory angiogenic functions of HUVECs induced by HG-triggered macrophages, HUVECs were co-incubated for 24 h with BMDMs pretreated with indicated administrations. Our findings revealed that the proportion of EdU + cells was increased after the addition of Nar to the HG niche, whereas a remarkable decrement under the treatment of Compound C was observed (Figure 4A). Meanwhile, the results from the wound scratch and transwell migration assay indicated that Nar administration markedly accelerated the rate of cell scratch closure and enhanced HUVEC mobilization to the lower chamber when compared to the HG stimulation alone, but these effects were inhibited by Compound C intervention (Figures 4B,C). In addition, there was an enhancement in the tube formation ability of HUVECs in the Nar group, which then was restrained in the Compound C group, as indicated by the number change in tube branch points (Figure 4D). In terms of the bioactive molecules involved in angiogenesis, we found that Compound C dramatically abolished the Nar-evoked expression elevation of PCNA, HIF-1α, MMP-1, and MMP-9, which acted as positive regulators of angiogenesis. The expressions of anti-angiogenic factors p21 and TIMP-1 were prohibited by Nar intervention but were obviously enhanced by Compound C (Figures 4E–G). To further investigate the molecular mechanisms required for angiogenesis in HUVECs, we observed that Nar administration markedly increased the expression of VEGFR2, followed by the activation of the PI3K/Akt axis and then the elevation of the HIF-1α level, yet this pathway was inhibited after Compound C intervention (Figure 4H). Our results suggested that Nar was likely to exhibit pro-angiogenic effects by switching the M1-related inflammatory niche to the M2-related regenerative microenvironment formed by macrophages.

### 3.5 Nar abated local inflammation and promoted diabetic wound repair *in vivo*

**FIGURE 5:**
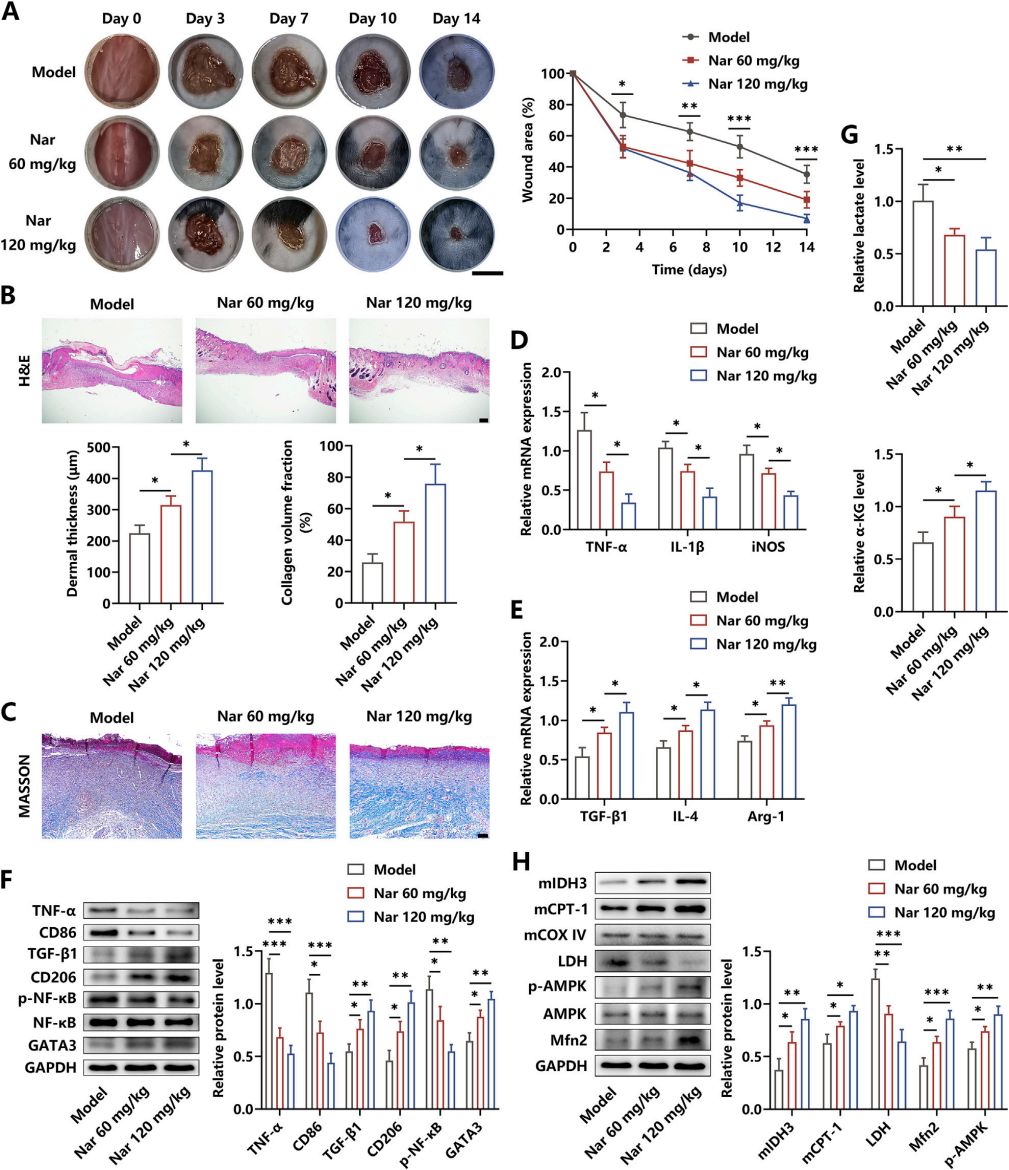
Nar treatment accelerated the healing processes of diabetic wound. **(A)** After the diabetic wound model was established, PBS, Nar at 60 mg/kg, or Nar at 120 mg/kg was locally injected for 14 days. The healing curve of each group is displayed (scale bar: 500 mm). **(B, C)** H&E and MASSON staining of the wound tissue in each group (scale bar: 200 μm **(B)** and 100 μm **(C)**). **(D, E)** The mRNA expression of inflammation-related molecules was measured using qRT-PCR. **(F)** The protein expression of inflammation-related molecules was measured using Western blot. **(G)** Nar dose-dependently decreased the level of lactate and increased the level of α-KG in the wound tissue. **(H)** The AMPK/Mfn2 pathway and expressions of LDH, mitochondrial CPT-1A, and mitochondrial IDH3 were detected using Western blot; n = 4; *p < 0.05, **p < 0.01, and ***p < 0.001.

A diabetic skin wound model was established to determine the therapeutic roles of Nar in inflammation elimination and damaged tissue healing. In this study, the diabetic mice were divided into three groups: PBS, low dose of Nar (60 mg/kg), and high dose of Nar (120 mg/kg) injection. The regenerative process of skin wound was recorded for 14 days, and we observed that Nar dose-dependently enhanced the rate of wound closure (Figure 5A). The histopathological detection showed that Nar treatment significantly promoted dermis regeneration in the wound area, and the extent of collagen deposition was strengthened after Nar administration in a dose-dependent manner (Figures 5B,C). Local injection of Nar suppressed the mRNA expressions of TNF-α, IL-1β, iNOS, and CD86 and facilitated the mRNA expressions of TGF-β1, IL-4, Arg-1, and CD206 in the HG microenvironment (Figures 5D–F). Meanwhile, the lactate level and NF-κB activity were decreased and the contents of α-KG and GATA3 were increased separately after Nar intervention in the wound tissue (Figures 5F,G). Moreover, the Western blot analysis suggested that Nar administration obviously induced AMPK activation and downstream Mfn2 expression, accompanied by metabolic reprogramming, as evidenced by LDH level reduction and mitochondrial IDH3 and CPT-1 content elevation (Figure 5H).

**FIGURE 6:**
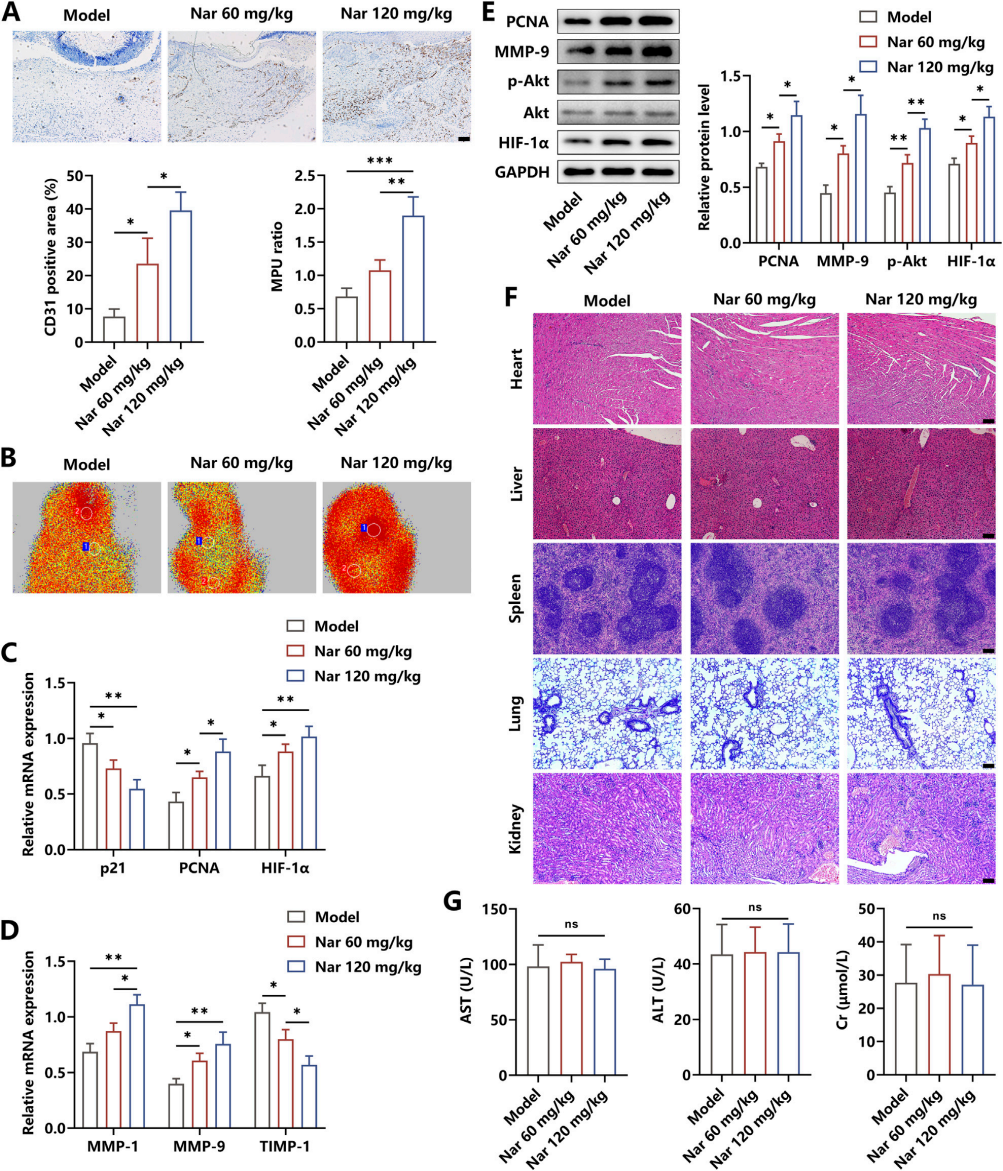
Effects of Nar on the angiogenesis and toxicity *in vivo*. **(A, B)** Immunohistochemical staining of CD31 and MPU ratios reflecting the blood flow in each group (scale bar: 100 μm). **(C–E)** The mRNA and protein expression of angiogenesis-related molecules were measured using qRT-PCR and Western blot. **(F,G)** The H&E staining of the heart, liver, spleen, lung, and kidney, along with blood levels of AST, ALT, and CK, in each group (scale bar: 100 μm); n = 4; *p < 0.05, **p < 0.01, and ***p < 0.001.

To further explore the effects of Nar on angiogenesis in the wound area, immunohistochemical detection was performed. As shown in Figure 6A, Nar dose-dependently increased the expression of CD31, which is an important biomarker of vessel tissues. The value of the MPU ratio, reflecting blood flow, was increased with the dose increment of Nar (Figure 6B). We found that Nar treatment enhanced the activity of the pro-angiogenic pathway and facilitated the expression of targeting effector molecules, as indicated by the results from qRT-PCR and Western blot (Figures 6C–E). Then, with the histopathological examination, we discovered that Nar treatment had no toxicity on the main organs *in vivo* (Figure 6F). Furthermore, findings from the blood biochemical test showed that Nar failed to alter the levels of AST, ALT, and Cr of mice (Figure 6G). Collectively, our results suggested that Nar exerted beneficial roles in promoting diabetic wound healing via attenuating local inflammation responses and subsequently favoring angiogenesis.

## 4 Discussion

There is evidence in the literature indicating that multiple pathogenic factors, particularly inflammation responses, exert detrimental effects on regulating the process of wound healing (Peña and Martin, 2024). Repressing the normal functions of endothelial cells, including proliferation and migration, inflammatory stimuli restrain the development of angiogenesis in the damaged tissue, thereby triggering the supply shortage of nutrients and oxygen, and finally leading to the delayed healing of diabetic wounds (Mao J. et al., 2025). Cumulative studies have depicted that the therapeutic effects of Nar are attributed to anti-inflammation, anti-oxidation, autophagy regulation, lipid metabolism, and more (Ri et al., 2023; Fang et al., 2023; Park et al., 2023; Wong et al., 2021). In this study, we discovered that Nar diminished HG-induced inflammation progression via regulating AMPK/Mfn2 axis-dependent metabolic reprogramming in macrophages, which in turn enhanced the angiogenic abilities of HUVECs and then facilitated the process of diabetic wound repair.

It is known that moderate inflammation response is beneficial for immunocytes to eliminate the invading bacterium and phagocytose cellular debris when exposed to tissue injury. Once the etiological factors persist in the local microenvironment, such as HG in the diabetic niche, the irritated immune cells continuously release pro-inflammatory cytokines and then induce inflammation extension, which result in cell death aggravation and tissue healing retardation, among which macrophages are considered the representative (Li et al., 2021; Sharifiaghdam et al., 2022). In the damaged tissue area, macrophages fail to switch from the M1 state to the M2 phenotype under the stimulation of diverse irritants, thereby inducing the amplification of M1 pro-inflammatory effects and diminution of M2 pro-regenerative effects, which contribute to the slow closure of diabetic wounds (Lu et al., 2024). As expected, our results manifested that HG significantly triggered macrophage polarization to the M1 phenotype, followed by the generation of inflammation cytokines, including TNF-α and IL-1β, which further activate inflammatory signaling cascades, verifying the pivotal pathogenic roles of HG in DFU development.

Traditional herbal medicines have been used for treating diseases and improving health for thousands of years, and the phytochemicals isolated from them are documented to possess therapeutic roles to some extent (Puri et al., 2023). Evidence from basic experiments has clarified that the bioactive agent Nar exhibits potent anti-inflammatory roles in abolishing aliment progression. Ri et al. reported that administration of Nar effectively inhibited NLRP3/IL-1β-related pyroptosis in LPS-primed macrophages through suppressing the inflammation signaling molecules NF-κB, MAPK, and PI3K/Akt (Ri et al., 2023). Likewise, Ha et al. revealed that the inflammatory proteins, including iNOS, COX-2, IL-1β, and TNF-α, in LPS-stimulated macrophages developed reduced expression after Nar intervention, which were partly attributed to drug-induced prohibition of NF-κB and MAPK pathways (Ha et al., 2012). Another study showed that Nar treatment restrained the expression of thioredoxin-interacting protein, followed by activation repression of NLRP3 inflammasome and alleviation of oxygen−glucose deprivation/reperfusion-induced damage (Luo et al., 2024). In this study, we observed that Nar markedly promoted macrophage repolarization from M1 to M2 status and enhanced the production of anti-inflammatory molecules TGF-β and IL-4, confirming the efficient effects of Nar on antagonizing inflammation development.

Considerable documentations have clarified that metabolic reprogramming frequently occur in tissue-resident macrophages, which affect the dynamic balance between local inflammatory effects and anti-inflammatory actions and determine the outcome of several diseases, including cancer, atherosclerosis, cardiomyopathy, and diabetes (Chen et al., 2022; Liu et al., 2023; Xiao et al., 2023; Russo et al., 2021). Glycolysis is defined as a glucose catabolism pathway that occurs in the cytoplasm for energy acquisition under hypoxic conditions, which is the main glucose metabolic mode in M1 macrophages. LDH, the rate-limiting enzyme of glycolysis, is responsible for the generation of lactate, which is the primary end product. It has been affirmed that under an aerobic situation, OXPHOS located in the mitochondria is regarded as the principal glucose degradation way for energy capture, and the key enzymes including CPT-1, IDH3, and SDH are involved in the mechanism. With respect to M2 macrophages, OXPHOS is the dominant intracellular glucose metabolic pathway, and several metabolites comprising α-KG, itaconate, and succinate are required for the pro-regenerative functions of the M2 phenotype (Liu et al., 2023; Wculek et al., 2023). Approaches aimed at inducing OXPHOS enhancement and glycolysis repression are demonstrated to be excellent in promoting anti-inflammation-related macrophage repolarization (Li et al., 2025; Bi et al., 2025). Here, we observed that Nar administration remarkably reduced the level of lactate and increased the content of α-KG *in vitro* and *in vivo*. Of note, in the Nar-treated groups, the expression of LDH was decreased, while there was an elevation in the protein content of mitochondrial CPT-1 and IDH3 without altering their transcriptional levels, suggesting that Nar probably affected mitochondrial homeostasis to regulate the location of rate-limiting enzymes and then mediate the progress of OXPHOS.

An ever-growing wealth of evidence illustrates that AMPK is believed to be a hub of metabolic control, which perceive falling cellular energy status by direct interactions with ATP, ADP, and AMP, elucidating the crucial roles of AMPK in the management of metabolic disorders like obesity, dyslipidemia, and diabetes (Herzig and Shaw, 2018; Lin and Hardie, 2018; Trefts and Shaw, 2021). Mfn2m located on the outer membrane of mitochondrionm is necessary to control the fusion and fission of the mitochondrial membrane, which is a prerequisite for proper functioning of the mitochondria. There were studies indicating that Mfn2 was capable of shortening the distance between mitochondria and sarco/endoplasmic reticulum and then enhancing their crosstalk, thereby contributing to optimize CD8^+^ T cell function and attenuate oxidative stress-induced endothelial cell injury (Yepuri et al., 2023; Yang et al., 2023). Recently, an intracellular signal cascade comprising AMPK and downstream Mfn2 was discovered to exert various pharmacological effects, including inflammation relief, reactive oxygen species elimination, autophagy mediation, and apoptosis inhibition (Zhang et al., 2021; Yu et al., 2021; Ashraf and Kumar, 2022; Hu et al., 2021). Our results revealed that Nar treatment increased the activity of the AMPK/Mfn2 axis, accompanied by OXPHOS enhancement and glycolysis diminution. Given that the AMPK/Mfn2 pathway was deeply implicated in the improvement of mitochondrial health (Trefts and Shaw, 2021), the underlying mechanism of Nar-triggered metabolic reprogramming was possibly that upregulation of Mfn2 maintained membrane homeostasis, reinforcing the stability of the inner mitochondrial microenvironment, which restored the activities of key enzymes in the tricarboxylic acid (TCA) cycle and then indirectly suppressed the initiation of glycolysis. In addition, we observed that Nar-induced inflammatory macrophage repolarization to M2 status was reversed by the inactivation of the AMPK/Mfn2 signal axis. An increase in NF-κB activity and its target gene expression and a decrease in anti-inflammatory GATA3 and cytokine expression were observed after Compound C or si-Mfn2 application. Considering that lactate was reported to be associated with NF-κB activation and metabolic intermediates in the TCA cycle were unveiled to be the upstream regulators for GATA3 expression (Faas et al., 2021; Arora et al., 2022), we inferred that Nar-related activation of the AMPK/Mfn2 cascade triggered the alteration of metabolite profiles, which then resulted in the priority of inhibitory signals in the inflammation regulation network in macrophages.

Evidence from laboratory statistics has established that the angiogenic abilities in the wound area play vital roles in determining the tissue repair speed (Guan et al., 2021; Chen et al., 2023). In the diabetic micro-niche, vascular endothelial cells, as the main executor of angiogenesis, develop diverse behaviors like early apoptosis, poor proliferation, and slow migration, which are detrimental to the angiogenic processes when exposed to harmful stimuli, especially inflammation (Wang et al., 2023). A large number of studies indicate that bioactive molecules like phytochemicals and small non-coding RNAs weakening local inflammation responses are advantageous to facilitate the angiogenic abilities of endothelial cells (Li et al., 2022; Wang G. et al., 2021). Here, our findings manifested that Nar obviously enhanced the proliferation and migration of HUVECs under the microenvironment of HG-evoked macrophages, which was repressed by AMPK inhibition. Moreover, the activity of the VEGFR2/PI3K/Akt/HIF-1α pathway, a growth-promoting signal axis (Wang Z. et al., 2021), was found to be increased after Nar administration but suppressed by AMPK inhibitor incubation. In view of the pro-inflammatory property of M1 and pro-regenerative capacity of M2 macrophages, Nar was likely to exert dual actions in favoring angiogenesis by abolishing M1-related inflammation cascades and enhancing M2-related proliferative pathways.

## 5 Conclusion

Taken together, we, for the first time, discovered that Nar promoted diabetic wound repair by inhibiting inflammation and facilitating angiogenesis. Through activating the AMPK/Mfn2 signal pathway, Nar effectively enhanced the extent of OXPHOS and repressed the activity of glycolysis, thereby contributing to macrophage repolarization from the M1 to M2 phenotype and then inducing the generation of pro-regenerative micro-niche-favoring angiogenesis. This study provided a scientific basis for the potential application of Nar in the management of DFU.

## Data Availability

The original contributions presented in the study are included in the article/supplementary material; further inquiries can be directed to the corresponding author.
